# Experiences of implementation of personalised risk estimates for breast cancer in clinical practice: a systematic review and qualitative synthesis

**DOI:** 10.1007/s12687-026-00918-3

**Published:** 2026-07-06

**Authors:** N. B. Fennell, S. Abukar, I. Kuhn, P. Linneker, C. Wilson, M. Tischkowitz, S. Archer

**Affiliations:** 1 Department of Genomic Medicine, University of Cambridge, Cambridge, CB2 0QQ UK; 2 School of Clinical Medicine, University of Cambridge, Cambridge, UK; 3 Medical Library, University of Cambridge, Cambridge, UK; 4 Department of Public Health and Primary Care, University of Cambridge, Cambridge, UK

**Keywords:** Breast cancer, Clinical implementation, Risk stratification, Breast screening, Systematic review

## Abstract

Breast cancer risk prediction tools are increasingly used in clinical practice to guide early detection, prevention, and shared decision-making. Unlike population-level screening, personalised risk estimates incorporate individual factors (family history, genetics, lifestyle, breast density), providing tailored assessments. These tools show promise for improving patient engagement and targeted prevention, but require effective implementation to ensure they enhance rather than complicate care. This review explores healthcare professionals’ experiences with providing personalised breast cancer risk estimates and women’s experiences of receiving them in clinical settings. Four online databases were searched for qualitative studies on the use of personalised risk estimates in clinical practice. Data were analysed using inductive thematic analysis. Seven papers were included; the majority based in the UK screening setting. Most used interview and focus-groups, with thematic analysis. Both healthcare professionals and women expressed high acceptance of personalised risk estimates. Women found the information empowering and useful for future health planning. Effective communication and prompt follow-up from healthcare professionals were crucial for positive experiences. Professionals highlighted challenges in implementation, including the need for additional healthcare professionals, safe care pathways, and technology. The studies focused on stratified breast screening, raising questions about offering less frequent screening to lower-risk women. This approach must be supported by strong evidence, and women should retain choice in screening intervals. Personalised risk estimates are favourably viewed in clinical practice, but studies mainly examined research settings. Further research is needed to understand real-world implementation, identify barriers, and optimise use across diverse clinical environments.

**Supplementary Information:**

The online version contains supplementary material available at https://doi.org/10.1007/s12687-026-00918-3.

## Introduction

In the UK, approximately 56,800 people are diagnosed with breast cancer (BC) annually. Despite 75% surviving for 10 years or more post diagnosis, 11,500 died between 2016 and 2018. The disease accounted for 15% of all new cancer cases between 2015 and 2017, making it the most common cancer in the UK (Cancer Research UK 2025). In 2021, the majority of BCs were diagnosed at stage 1 (43%) or stage 2 (41%) (CRUK Cancer Intelligence 2025). In 2024, the economic cost of BC in the UK was estimated to be between £2.6 and £2.8 billion, potentially rising to £3.4 billion by 2034 (Bush et al. 2024).

Hereditary forms of BC account for approximately 6% of all BC diagnoses (Rowlands et al. 2024). Germline and somatic genetic testing can influence treatment and management options, as well as future risk of additional cancers. Should a germline pathogenic variant (GPV) be detected, family members may be offered predictive genetic testing to enable early detection or prevention.

In the UK, genetic testing for BC is guided by the National Genomic Test Directory, which sets out who may be offered testing, and under what circumstances (England NHS 2025). Management of women with a moderate or high risk of BC is guided by the National Institute for Health and Care Excellence (NICE) guidelines CG164 which may involve additional surveillance, risk reducing medication and risk reducing surgery (National Institute for Health and Care Excellence 2023).

The use of multifactorial risk assessment tools (MRATs) is becoming more routine in clinical BC care and prevention. These tools are based on risk-prediction models, such as BOADICEA (Breast and Ovarian Analysis of Disease Incidence and Carrier Estimation Algorithm) or Tyrer-Cuzick, which combine genetic and non-genetic factors to calculate risk of breast and other cancers and the probability of an individual carrying a GPV (Lee et al. 2019; Tyrer et al. 2004).

Calculating a women’s personalised BC risk estimate may also allow stratified screening; high-risk women may be screened more frequently, while low-risk women may have longer intervals. According to NICE guidelines (CG164), women are categorised into one of three risk thresholds for BC; near-population (up to 17%), moderate (17–30%) and high (30% or greater) (National Institute for Health and Care Excellence 2023). Those at population risk are offered three-yearly screening from the age of 50 through the NHS Breast Screening Programme (NHSBSP). Moderate risk women may be offered additional screening between the ages of 40 and 50, and chemoprevention. High-risk women’s options depend on age and GPV status, but may include surgery to manage their breast/ovarian cancer risk, earlier screening and chemoprevention, outlined in NICE guideline CG164 (National Institute for Health and Care Excellence 2023).

Several studies have assessed the validity of personalised risk estimates, and the acceptability of their use in prospective, non-clinical contexts through implementation science frameworks, such as the Consolidated Framework for Implementation Research (CFIR—which evaluates the relationship between the intervention, inner and outer settings, and stakeholders (Damschroder et al. 2022)) have shown promising results. Other studies focusing on risk communication and stratified screening have confirmed the importance on how risk information is framed, interpreted and acted upon (Woof et al. 2020). However, very little research has explored user (i.e.healthcare professionals (HCPs) and women’s) experiences of personalised risk estimates in a clinical setting. Given the addition of such tools to the NICE guidelines CG164, as well as the development of consensus guidelines (ABS/UKCGG/CanGene-CanVar) (National Institute for Health and Care Excellence 2023; Bellhouse et al. 2021; Tsoulaki et al. 2024), a review of the evidence is urgently needed The aim of this systematic review is to collate and analyse studies which qualitatively investigate the experiences of both HCPs and women on the implementation of personalised risk estimates within a clinical setting.

## Methods

The protocol and reporting for this systematic review was guided by the Preferred Items for Systematic Reviews and Meta-Analysis (PRISMA) 2020 (Page et al. 2021). The review was registered on PROSPERO (CRD42024581170) before searches were conducted (PROSPERO 2024).

### Search strategy

The research questions and search strategy were informed by a modified PICOS framework (Population, Intervention, Comparison, Outcome, Study Type), focusing on women and HCPs, and their experiences of personalised risk estimates in a clinical setting using qualitative research (Richardson et al. 1995). Searches were designed and trialled on Embase with the assistance of a research librarian (IK), then translated into Medline, PsycINFO and CINAHL. A combination of key words and subject headings (eg MeSH) related to the research question were used to search for titles and abstracts. Search facets were based on BC, personalised risk estimates and genetics (see supplementary file 1). Papers were assed for their use of qualitative methods at the title/abstract and full text screening stages to ensure that relevant papers that had a qualitative element could be identified and included where appropriate. Date restrictions for publications were not imposed due to the recent nature of the research in question. Publications from any country were deemed eligible, however due to resource constraints, only articles available in English were included.

Searches were carried out on all four databases on 21 st November 2024 and exported to Endnote.

### Study selection

Inclusion and exclusion criteria to address the research question is shown in Table 1.

**Table 1:** Inclusion and Exclusion Criteria

Inclusion Criteria	Exclusion Criteria
- Women aged 18 and over	- Men & transgender individuals
- Any races/ethnicity	- Women younger than 18
- Women seeking risk assessment for breast cancer in a clinical setting	- Women who received a personalised risk estimate for anything other than breast cancer
- Healthcare professionals or clinical support staff who provide breast cancer personalised risk estimate using a clinical tool (e.g. CanRisk)	- Women who were provided with a personalised risk estimate in a non-clinical setting
- Women who were provided with a personalised risk assessment as part of a breast cancer risk assessment, by a healthcare professional	- Women with a previous diagnosis of breast cancer
- Having a qualitative element*	- Quantitative-only research studies

*—the use of qualitative elements was assessed during title and abstract screening to ensure a thorough search of the literature, and to avoid missing relevant publications

One reviewer (NF) conducted the database searches, removed any duplications, and conducted primary title and abstract screening. Duplicates were removed using EndNote 20 (The EndNote Team 2013). Remaining duplicates were removed manually in Rayyan during title and abstract screening. Secondary review of titles and abstracts were conducted by reviewers PL, CW, SAb and SAr, each screening approximately 10% of articles. Discrepancies were discussed as a group and a consensus was obtained. Cohen's Kappa was used to calculate interrater reliability. The review team were comprised of a mix of experience and backgrounds, ranging from medical students (SAb and PL) to an experienced clinical researcher (SAr). Regular meetings provided opportunities for team members to reflexively discuss their approach and how their subjectivities may have shaped their outlook and decision making when reviewing the papers.

### Quality assessment

The Critical Appraisal Skills Programme (CASP) checklist for Qualitative Research was used for quality assessment of the articles used (Critical Appraisal Skills Programme 2024). All articles were assessed by NF and PL independently. No articles were excluded based on quality.

### Data extraction & synthesis

Descriptive information was extracted by reviewer NF into a data extraction form, including study design, analysis, number of participants, location, and risk assessment model. The results sections were extracted into a separate document. Descriptive analysis was conducted on study-related data. The qualitative results were analysed by inductive thematic analysis using Braun and Clark’s approach (Braun and Clarke 2006); a realist epistemology was adopted. This methodology and epistemology was chosen to enable researchers to effectively collate, thematically organise and describe women’s and HCP’s experiences using secondary data. Coding was conducted by NF using Nvivo 14 and developed into candidate themes, which were discussed and refined with SAr.

## Results

### Study selection

A total of 6064 articles were imported into EndNote 20. After duplicates were removed, 4,163 articles were imported into Rayyan. A further 588 duplicate articles were removed manually, leaving 3,575 articles left for title and abstract screening.

**Table:** 

Title & Year	Country	Research Study	Study Design	Participants	Risk Model Used
The feasibility of implementing risk stratification into a national breast cancer screening programme: a focus group study investigating the perspectives of healthcare personnel responsible for delivery (French et al. 2022) 2022	UK	BC-Predict	Focus Group	Healthcare Professionals	Tyrer-Cuzick (v8)
Healthcare professionals’ views following implementation of risk stratification into a national breast cancer screening programme (Hawkins et al. 2022) 2022	UK	BC-Predict	Semi Structured Interviews	Healthcare Professionals	Tyrer-Cuzick (v8)
Canadian Healthcare Professionals’ Views and Attitudes toward Risk-Stratified Breast Cancer Screening (Lapointe et al. 2023) 2023	Canada		Surveys but contained open ended questions	Healthcare Professionals	n/a
Women’s experiences of risk-stratified breast cancer screening in the MyPeBS trial: a qualitative comparative study across two European countries (McWilliams et al. 2026) 2024	UK/France	MyPEBS	Semi Structured Interviews	Women	Mammorisk or Tyrer-Cuzick (v8)
What do women think about having received their breast cancer risk as part of a risk-stratified NHS Breast Screening Programme? (McWilliams et al. 2023) 2023	UK	BC-Predict	Telephone interviews	Women	Tyrer-Cuzick (v8)
Introducing a low-risk breast screening pathway into the NHS Breast Screening Programme: Views from healthcare professionals who are delivering risk-stratified screeni2021	UK	BC-Predict	Focus Group or Telephone Interview	Healthcare Professionals	Tyrer-Cuzick (v8)
2021	UK	BC-Predict	Interviews	Women	Tyrer-Cuzick (v8)

After title and abstract screening, 62 studies were included for full text review. Due to the low number remaining after title and abstract screening (1.5% of articles were included), Kappa’s Coefficient for authors CW, SAb and PL were low (< 0.35). A higher Kappa’s Coefficient was obtained with author SAr (0.75) as this subset contained more “included” articles. Team discussion around approaches to screening did not suggest significant difference across the team.

**Fig. 1:**
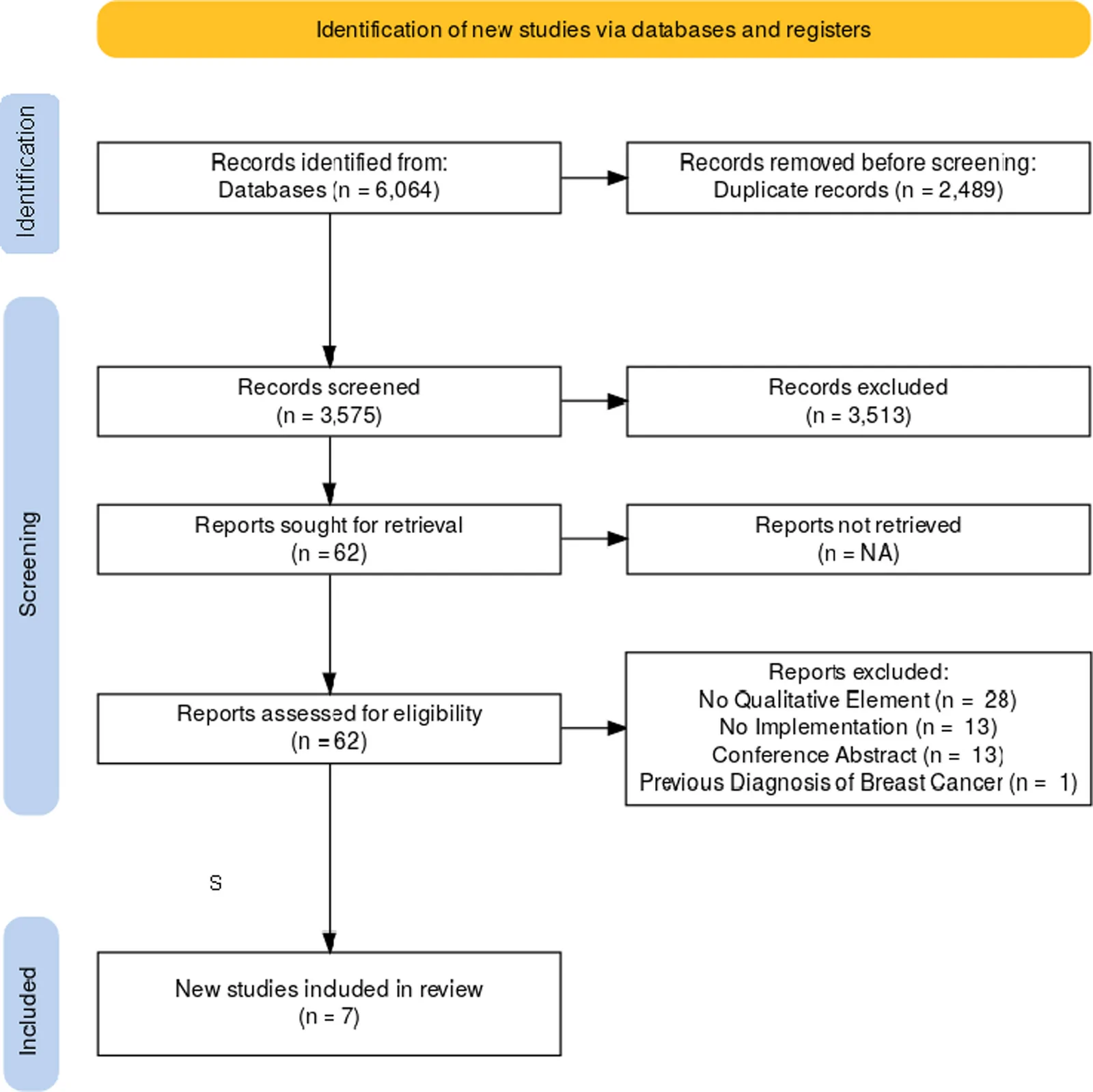
PRISMA flow for articles in this Systematic Review

Of the 62 studies, 55 were excluded after full-text screening, mainly because they lacked a qualitative element, did not implement personalised risk assessment in a clinical setting, or were conference or poster abstracts (see PRISMA in Fig. 1).

A total of seven papers were included after full text screening, all published between 2021–2024. Four papers focused on HCP experiences of implementing multifactorial risk assessments (French et al. 2022; Lapointe et al. 2023; Woof et al. 2021; Hawkins et al. 2022), and three focused on women’s experiences (McWilliams et al. 2021, 2023, 2026). Studies ranged from 14 to 61 participants/respondents. For the purposes of this review, patient and staff experiences are reported separately.

The majority of the studies were conducted solely in the UK (5/7) (French et al. 2022; McWilliams et al. 2021, 2023; Woof et al. 2021; Hawkins et al. 2022). One was a joint study between the UK and France (McWilliams et al. 2026), and the other was based in Canada (Lapointe et al. 2023). Women involved in the study were aged 40–74. HCPs had a variety of clinical roles and levels of seniority, ranging from trainees to clinical fellows and consultants. HCPs were mostly based in secondary/screening services.

Patient participants were predominately white British or Irish, making up 100%, 90% and 82% of the study cohorts (McWilliams et al. 2021, 2023, 2026).

Both the MyPeBS and BC-Predict studies used Tyrer-Cuzick to calculate personalised risk estimates. MyPeBS collected details on risk-modifying factors through baseline self-reported surveys, alongside mammographic density data from screening, and polygenic score (based on the Mavaddat 313 panel (Mavaddat et al. 2019)). BC-Predict collected risk-modifying factors via self-reported questionnaires, and mammographic density when available. This study included PRS in a subset of participants (Gareth Evans et al. 2023). How results were returned to participants varied, even within studies, including letters, emails, and face-to-face. MyPeBS used 5-year BC risk, whereas BC-Predict used 10-year risk.

Table 2 summarises risk categories assigned to participants in studies 4, 5 and 7, noting that study 7 only recruited those in the low-risk category (McWilliams et al. 2021, 2023, 2026).

**Table 2:** Breakdown of Risk Categories assigned to participants in studies 4, 5 and 7 (McWilliams et al. 2021, 2023, 2026)

Risk Category	N = total number of participants			
	Study 4	Study 5	Study 7	Total
Low	17	10	23	50
Average	13	9	0	22
Moderate (High)	14	11	0	25
High (Very High)	8	10	0	18

Six out of seven studies used focus groups or semi-structured interviews to collect data (French et al. 2022; McWilliams et al. 2021, 2023, 2026; Woof et al. 2021; Hawkins et al. 2022). The seventh study was survey-based, but contained open ended questions assessing experiences of the use of the risk assessment tool (Lapointe et al. 2023). All studies used thematic analysis (French et al. 2022; Lapointe et al. 2023; McWilliams et al. 2021, 2023, 2026; Woof et al. 2021; Hawkins et al. 2022).

## Patient results

Women’s experiences of personalised risk assessments and risk stratification focused around two main themes, each containing several subthemes;

(i) Acceptability, Engagement and Reactions to Personalised Risk Estimates and Stratification
(ii) Implications on Risk Management and Future Health.

### Theme 1: Acceptability, engagement and reactions to personalised risk estimates and stratification

#### Subtheme 1: Engaging with studies

Women appreciated the invitation to receive a personalised risk estimate and the future health information it provided (McWilliams et al. 2023). They viewed this as empowering, giving them a greater sense of control over their future health;

*‘If you don’t know what your risk is, you could just carry on and, you know, just end up getting something like breast cancer and it be a complete shock to you.’ (Erica, High)* (McWilliams et al. 2023)

For some women, study participation was emotionally triggering, reminding them of past family experiences of the disease, as well as future cancer risk (McWilliams et al. 2023). Waiting for results did not appear to negatively impact participants, with many reporting that they did not think about their BC risk whilst awaiting results (McWilliams et al. 2023).

#### Subtheme 2: Women’s reactions to results – moderate and high risk

Risk assessments were returned to participants through letters and face-to-face or phone consultations. MyPebs study results, sent by letter and followed by phone calls, were received positively and helped understanding. It also provided an opportunity to ask questions and advice regarding their future risk. Some women found it helpful to know their result before this consultation, as it allowed them to psychologically prepare and gave time to process the information. However, others found it emotionally difficult to find out their high- or very high-risk status without HCP support, quoting:

‘I think it would have been better, less of a shock, to have the two things the other way round, so to speak to a doctor and have any questions answered immediately. (Gillian, Very High) (McWilliams et al. 2026).

Prompt HCP contact was beneficial to women after receiving results, particularly for those assessed as being at high- or very high-risk. One high-risk woman, upon opening her results letter, had difficulties processing her results due to emotional triggers and subsequent fears about future health. This was dissipated once she ‘*spoke to the [HCP] about it’* after which she *‘was absolutely fine’,* however, this was following ‘*two weeks mulling it over at home and perhaps blowing things out of proportion’ (Ruby, High) (*McWilliams et al. 2023*).*

Misunderstanding arose in one case where high-risk results were communicated by letter without follow-up from a HCP. The participant reported believing she ‘*was going to get cancer in the next five years’ (Anne-Marie, Very-High)* (McWilliams et al. 2026).

#### Subtheme 3: Women’s reactions to results – low and average risk

For low- or average-risk women, HCP contact post-results did not seem as critical, with one participant stating her hospital visit to receive an average-risk result as unnecessary; *‘I came specifically for something that wasn’t a big deal’ (Armelle, Average)* (McWilliams et al. 2026). This was mirrored for participants where the results aligned with what they expected and were not “out of the blue” (McWilliams et al. 2023). Many who received an assessment categorising them as low-risk did not recall specific details contained within their letter, however remembered that the results were ‘good news’ (McWilliams et al. 2026).

#### Subtheme 4: Format of risk communication (letters)

Detailed letters were appreciated by several participants as it allowed them to go back to the results when they needed, aiding in understanding –

*‘I read through the rest of it, put it down and went back to it again later on, I took it in a bit more, and I thought, oh right, okay, I can see why I’m falling into that [high-risk category]’ (Faye, High) (*McWilliams et al. 2023*).*

Letter structure and content was reported as an important factor for both retention of knowledge and psychological impact of results. Presenting information in different formats (e.g. statistical presentation of risk, infographics) was helpful for women, especially when going on to discuss their risk with a HCP, which reduced temporary psychological distress (McWilliams et al. 2023). The framing of the impact of modifiable and non-modifiable risk factors was triggering for some women, leading to disempowerment and the feeling of being judged;

*“The fact that I’d not had children till I was a bit older, […] knowing that risk factor isn’t very much help to me really, ‘cause there’s nothing I can do about it.” (Anita, Moderate) (*McWilliams et al. 2023*).*

#### Subtheme 5: Prior assumptions of risk

Prior assumption of risk tended to influence the experience of receiving a personalised risk assessment, shaping emotional reactions and interpretation of results. Risk assessments were considered the most reassuring when it matched a woman’s prior expectation of risk, especially when this was low- or average-risk. Mostly this tracked with a woman’s lived experience of cancer, however incongruity was noted when a woman’s actual risk, usually lower, did not align with a high-perceived risk due to family history. One woman, assessed as low-risk, did not feel that she could incorporate her result into her health narrative due to a previous cancer death in the family (McWilliams et al. 2023).

*‘…But I still, probably in my mind think, well surely that’s not correct. And I should just accept it, shouldn’t I, but I think because having gone through losing family members...’ (Hannah, Low) (*McWilliams et al. 2023*).*

Prior risk assumptions fed into women’s belief in their results, particularly when they saw inconsistencies between lived experiences and HCP advice, or between HCP advice and risk assessment results. This was the case for one participant, who received a high-risk assessment, despite the HCP being reassuring about the diagnoses of BC in the family being at a later age (McWilliams et al. 2026). Another participant disregarded her results due family members being diagnosed with BC despite practicing the healthy lifestyle guidelines cited in her results letter (McWilliams et al. 2023, 2026).

#### Subtheme 6: Belief in results

Belief in results tracked with trust in the HCP providing their risk assessment, and having a pre-existing relationship with the HCP was highly appreciated. Furthermore, this facilitated acceptance of results and gave women more confidence in advice regarding risk management (McWilliams et al. 2026). When discussing the option of receiving less frequent screening after a low- or average risk assessment, one woman quoted:

*‘...if the NHS feel confident in giving that advice then I would feel confident in taking because you are a trusted supplier of messages’ (*McWilliams et al. 2021*).*

The need for women’s trust in HCPs is further highlighted by a participant who was given her result by her primary care doctor, who was unable to explain why she had received a very high-risk assessment. This led to an upsetting experience, and her questioning whether she believed her result (McWilliams et al. 2026). Women were also left unsettled when thinking about how their HCP may react to their risk assessment, leading to feeling alone when considering risk management options. One woman thought her doctor would judge her for her high-risk result, and was fearful she’d be told to *‘go away and lose some weight’,* rather than being prescribed chemoprevention, as desired (McWilliams et al. 2023).

### Theme 2: Implications for risk management & future health

Women receiving a personalised risk estimate described it having a favourable impact on views on future health and autonomy. Some women felt it was their duty to take responsibility for their own health, and felt being able to access risk personalisation would aid in this;

*‘…knowledge is power and, you know, we all need to take responsibility for our own health and if we know in advance then we can deal with it’ (Abigail, Average) (*Aldila et al. 2024*).*

#### Subtheme 1: Lower-risk results

Women who were low- or average-risk were left with a reassuring outlook on future health, and was quoted as being *‘one less thing to worry about’* (McWilliams et al. 2023). One participant admitted to feeling more comfortable taking HRT now that she knew that her BC risk was low, and that the medication would not be adding additional risk to an already increased risk (McWilliams et al. 2021).

Many with a low risk felt that although this was comparatively lower than others, they would remain vigilant, as they knew that low risk does not equal no risk:

*‘It could happen at any time so it’s not that I’m never going to get it, it’s just that I’m at, sort of, the low-risk end of the scale’ (*McWilliams et al. 2021*).*

When discussing the option of being offered low-risk screening, with longer intervals compared to standard NHSBSP (i.e. 5-yearly, compared to 3-yearly as per NHSBSP), women were of mixed opinions, stating that choice would be a major factor in acceptability. Many viewed the longer period between screening as worrisome, as this is ‘*where things can go awry’ (Constance, Low),* however some women were more accepting due to altruistic reasons, citing that resources being allocated to those at higher risk would be important;

*“The fact that I’m low-risk, so if they said to me, right, you’re not having any more mammograms, and I know that I’m lowest risk, then I’m quite happy with that because then it frees up NHS money for somebody who’s younger that, perhaps, does need it.” (Tracey, Low)* (McWilliams et al. 2026).

#### Subtheme 2: Screening intervals

Women may view this more favourably if given the option to extend their screening interval or delay screening, with the ability to return to regular screening if desired. (McWilliams et al. 2021):

*‘If I was given the choice and made my own decision based on more accurate details and facts and then that would be my decision, rather than every three years, then I’d be happier with that than just being told ‘you’re in that category, we’re going for every five years’ (*McWilliams et al. 2021*).*

As cancer communication often emphasises the benefits of early detection, women expressed a desire for clear, evidence-based guidelines and recommendations to be available on request; (McWilliams et al. 2026).

*‘… the study has to come up with some really good evidence bases behind it to change the mindset of us all ‘cause at some point somebody said, it’s clinically right to do it at three years’. (Angela, Low) (*McWilliams et al. 2026*).*

This was especially important to those who saw the longer screening intervals as a money-saving exercise, rather than evidence-based clinical practice (McWilliams et al. 2021).

#### Subtheme 3: Risk modifying behaviour

Women who received a personalised risk estimate were noted to be more open to discuss risk-modifiable factors with a HCP, such as weight and alcohol consumption (McWilliams et al. 2026). However, some women reported that these modifications were hard to maintain as part of a busy life, and were concerned about how these might influence future risk, if a healthy lifestyle was not adhered to (McWilliams et al. 2021). Women already practicing healthy living found conversations around risk-reducing medication (chemoprevention) helpful, as they felt it was something that could be managed (McWilliams et al. 2026).

### Health care professionals results

HCP experiences of the implementation of personalised risk assessments centred on two themes, and contained several subthemes;

- 1) Professional and Public Buy-In.
- 2) Capacity for Implementation and Future Management.

### Theme 1: Professional and public buy-in

Across studies, HCPs viewed personalised risk assessments and risk-stratified screening positively. The approach was considered a logical method for tailoring screening, empowering women to make informed choices, and reducing over-screening of those at lower risk (Lapointe et al. 2023; Woof et al. 2021; Hawkins et al. 2022).

*I think the idea is really good… We concentrate then on getting those ones that are high risk in. I mean, it makes sense, doesn’t it? (Superintendent Radiographer) (*Woof et al. 2021*)*

#### Subtheme 1: Reactions to high-risk results

While high-risk results could cause anxiety, this was considered a normal reaction that could be mitigated by clear management pathways. Reactions were seen as linked to pre-existing anxiety rather than a barrier to implementation (Hawkins et al. 2022). Multidisciplinary meetings were emphasised as essential during early implementation to ensure safety and stakeholder input (Hawkins et al. 2022).

#### Subtheme 2: Guidelines

Current guidelines for managing increased risk were viewed as inconsistent and unclear, creating a disconnect in national implementation (French et al. 2022). Participants called for clear management guidelines, robust screening pathways, and improved digital infrastructure to support efficient implementation (Lapointe et al. 2023; Hawkins et al. 2022). Guidance on prescribing preventive medications was also lacking (French et al. 2022). Establishing information-sharing systems to access women’s risk factors across services was suggested to enhance efficiency (Hawkins et al. 2022).

#### Subtheme 3: Media coverage and public engagement

Public understanding and acceptance were recognised as critical, particularly if screening intervals were extended for low-risk women (Woof et al. 2021). Clear, balanced media communication was viewed as key to public trust. Engaging underrepresented populations (e.g. ethnic minorities, low socioeconomic status, those with learning difficulties) from the outset was seen as vital to prevent widening health inequalities (French et al. 2022; Hawkins et al. 2022).

*“We need to be able to communicate […] to all the women […] not just the English-speaking ones…” (Mammography Manager) (*Hawkins et al. 2022*).*

#### Subtheme 4: Disengagement from screening services

HCPs expressed concern that women receiving low- or average-risk results may use them as justification for disengaging with screening. Open discussions were recommended to address misconceptions and support women make informed health choices (French et al. 2022*).*

### Theme 2: Capacity for implementation and future management

Many HCPs (screening managers, radiographers, risk consultations staff) thought risk-based screening would be feasible, seeing as many risk factors are already discussed during consultations.

*“…it was part of the interview process that we do with every patient anyway and there’s always going to be extra questions...” (Mammography manager) (*Hawkins et al. 2022*).*

### Theme 1: Staffing needs

Adequate staffing was viewed as critical for successful integration (French et al. 2022; Hawkins et al. 2022). Screening services and family history clinics are already under pressure, raising concerns about increased workload and burnout (French et al. 2022; Hawkins et al. 2022). Additional specialist staff would be required, including helpline operators and breast screening nurses (Woof et al. 2021). It was highlighted that this should not be automatically deferred to general practitioners (GPs), due to limited capacity and a need for specialist care (Lapointe et al. 2023).

Short clinical appointments, particularly in the GP setting (average appointments lasting only 9.2 min) were not seen as sufficient for data collection or thorough risk discussion (French et al. 2022; Salisbury 2019). Dedicated staff members who can coordinate and facilitate women undergoing this process were seen as essential.

Consistent infrastructure and guidelines across services were also emphasised, as requirements for implementation would vastly differ between sites (Hawkins et al. 2022).

### Theme 2: Confidence in results

Confidence in the accuracy and management of risk results was seen as vital, particularly for women classified as low risk who may receive less frequent screening. HCPs questioned how often reassessments should occur, given that risk factors (e.g., breast density, parity, family history) can change over time. A dedicated reassessment service was suggested (Woof et al. 2021).

*‘I’m just thinking about those who might think, right, okay, I’ve got a low-risk, but what if circumstances change? And sometimes they might have breast cancer in the family and they might not know, because a lot of women don’t tell. (Cancer Screening Improvement Lead) (*Woof et al. 2021*).*

### Theme 3: Low-risk screening

HCPs were concerned that women might misinterpret low-risk results as “no risk,” leading to reduced engagement in screening or self-checking (Woof et al. 2021):

*“… they might think, oh, I won’t get breast cancer because I’m such a low-risk..”. (Advanced Practitioner – Mammography) (*Woof et al. 2021*).*

They stressed the need for clear, balanced communication to maintain vigilance. Some expressed discomfort with recommending longer screening intervals due to fear of missed interval cancers.

*“I wouldn’t feel comfortable in telling somebody to have a longer gap in the screening if I wasn’t 100 per cent that [...] I personally wouldn’t be like, well, yeah, just leave it five years because I’d be really conscious of them developing a cancer in between. (Mammographer) (*Woof et al. 2021*)*

Offering women choice regarding screening frequency was viewed as important to sustain reassurance and trust. However, information must be tailored to avoid overwhelm (Woof et al. 2021).

## Discussion

Across the studies included in this review, the use of personalised risk estimates in a clinical setting were generally acceptable to both patients and HCPs, although these views may not reflect more diverse or underserved populations.

Women valued the opportunity for personalised care the insight into future risk. Although some women found waiting for results difficult, HCPs theorised that this would have been the case regardless of study participation. Whilst letters were generally acceptable for communicating results, prompt follow-up from a known HCP facilitated understanding and psychosocial adjustment, particularly for those at higher risk. This was exemplified by several women who either misunderstood or had an adverse emotional reaction to their results without having prompt HCP follow up. Clear letter content and structure, as well as trust in the HCP, were important factors in processing results, and should be considered when pathways and guidelines are being developed.

Although some HCPs worried about disengagement of low- or average- risk women, many participants understood that low risk did not mean no risk and remained vigilant. Clear guidelines, pathways, and effective communication were suggested to maintain engagement. HCPs highlighted additional personnel, technological infrastructure, and robust guidelines as requirements for successful implementation.

A key concern of both cohorts was screening intervals for low-risk women. Women emphasised that patient choice was key. Allowing women to decide whether intervals are extended or remain on standard schedules, leading to confidence in safety and efficacy. Research into extended intervals is ongoing, but consensus is lacking meaning this is a key area for future research before these risk estimates could be rolled out safely (Kelley-Jones et al. 2024; Ravesteyn et al. 2021; Kelley Jones et al. 2024). Control of media communication was also noted as important to ensure accurate public understanding.

Consensus guidelines exist for risk assessment tools such as CanRisk, but these do not focus on risk stratification, and implementation varies across sites (Tsoulaki et al. 2024). Comprehensive, consistently applied guidelines are needed to promote equitable care. The findings from this review suggest that guidelines (such as NICE CG164) could be strengthened by more explicit recommendations on implementation pathways, staffing needs and screening intervals, particularly for low-risk women.

Most studies involved women already engaged with healthcare services, highlighting gaps in addressing health inequalities for underserved populations. Participants were predominantly white, emphasising the need for research in more diverse populations and settings, including primary care and community outreach.

At the time of this review, much of the research came from the BC-Predict study. Ongoing studies, such as Precision HBOC (UK/US) and PRiMo (Australia), will broaden understanding of implementation experiences and acceptability (McInerny et al. 2024; Archer et al. 2022). Increased integration of AI and technology, for example through patient-facing apps used in the CanRisk-ClinGen study, may reduce clinician workload and improve implementation (CanRisk-ClinGen, n.d.).

HCP-focused studies primarily reflected screening services, with limited input from primary care or tertiary genetics services. Future research should capture these perspectives and explore the experiences of receiving personalised risk assessments outside of screening programs.

In conclusion, the findings of this review reinforce that implementation successful is shaped by the intervention itself, inner- and outer-setting factors, and the process through which it is introduced in line with the CFIR. In practice, this review has highlighted the need for (1) Structured communication pathways and guidelines, especially for high-risk results, (2) Clear, evidence-based communication about lengthening screening intervals for low-risk women (3) Additional specialised staff and improved IT infrastructure to support implementation, and (4) Proactive engagement with underserved communities to guide equitable implementation.

## Supplementary Information

Below is the link to the electronic supplementary material.Supplementary file1 (DOCX 341 KB)

## Data Availability

Articles used in this review are openly available (DOIs included in References). Search strategies available in Supplementary Information.

## Declarations

This article does not contain any studies with human or animal subjects performed by the any of the authors.
